# Supplementary material for: Characteristics of Gut Microbiota in Sows and Their Relationship with Apparent Nutrient Digestibility
Source: Int J Mol Sci. 2019 Feb 18;20(4):870. doi: 10.3390/ijms20040870 (PMC6412398; doi:10.3390/ijms20040870)
Supplement: Supplementary file 1 [file ijms-20-00870-s001.pdf]

Table S1 Information of the studies cited.

References	Age of pigs	Samples	Target	Sequencing technology
Hyeun BK et al. (2012)	10-22 weeks	Pig feces	V3	Roche 454
Zhao WJ et al. (2005)	1-6 months	Pig gut contents	V4	Illumina Miseq
Looft T et al. (2014)	3 months	Pig gut contents	V1-V3	Roche 454
Hyeun BK et al. (2011)	10-22 weeks	Pig feces	V3	Roche 454
Lu XM et al. (2005)	Piglet and adult pig	Pig manure	V1–V3	Roche 454
Lamendella R et al. (2005)	6 months	Pig feces	-	Roche 454
Whitehead TR et al. (2005)	Feeder pigs	Pig feces	-	-
Yatsunenko T et al. (2012)	0-18 years	Human feces	V4	Roche 454
Ye L et al. (2005)	-	Water	V4	Roche 454

Table S2 Ingredient composition (g/kg) of the experimental diet.

Diet composition	
Corn	560
Soybean meal	140
Wheat bran	260
De-mold agent	1
Vitamin A	2.4×10^{-3}-3.6×10^{-3}
Vitamin D3	5.5×10^{-5}-1.0×10^{-4}
Vitamin E	$\geq0.9\times10^{-3}$
Vitamin K3	4.5×10^{-5}-8.5×10^{-5}
Vitamin B1	$\geq7.0\times10^{-5}$
Vitamin B2	$\geq1.75\times10^{-5}$
Vitamin B6	$\geq1.0\times10^{-4}$
Vitamin B12	$\geq7.0\times10^{-7}$
Niacin	$\geq1.0\times10^{-4}$
Calcium Pantothenate	$\geq0.43\times10^{-4}$
Folat	$\geq3.5\times10^{-5}$

bBiotin	≥8.0×10^{-5}
Choline Chloride	≥8.0×10^{-3}
Copper	5.0×10^{-4}-8.75×10^{-4}
Iron	3.0×10^{-3}-5.0×10^{-3}
Manganese	1.2×10^{-3}-2.0×10^{-3}
Zinc	2.5×10^{-3}-3.75×10^{-3}
Iodine	1.8×10^{-5}-3.5×10^{-5}
Selenium	7.0×10^{-6}-1.2×10^{-6}
Calcium	5.6-7.2
Total Phosphorus (TP)	≥1.0
Salt	3.2%-4.4%
Moisture	≤4

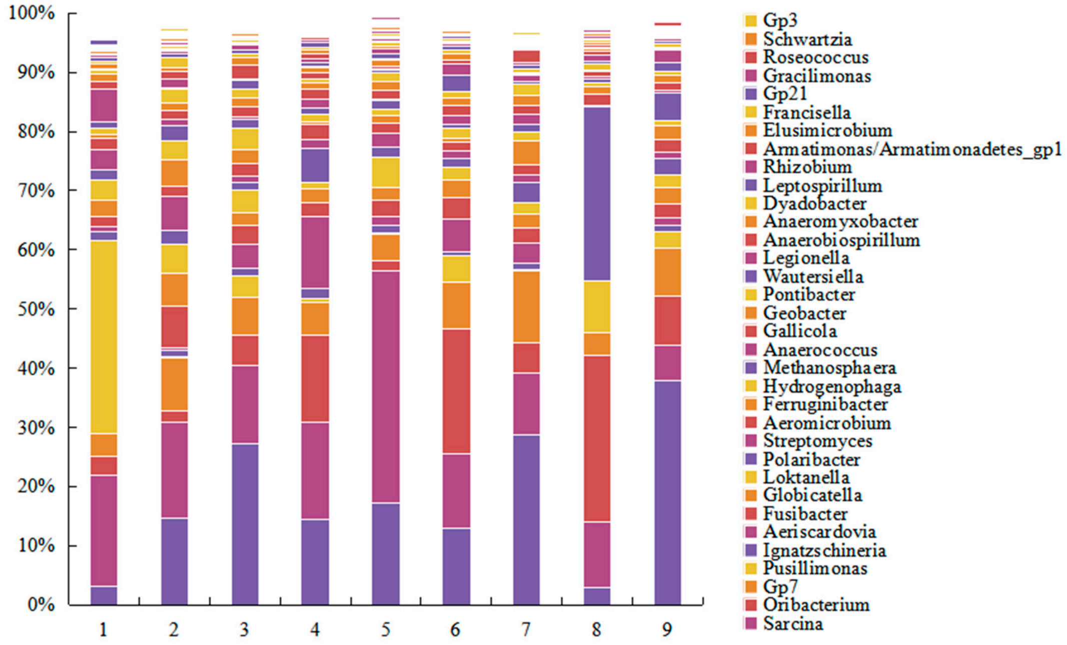

Fig. S1 Genus distribution of gut microbiota. Distribution of the genus as a percentage of the total number of identified 16S rRNA sequences from fecal samples of the 9 sows.

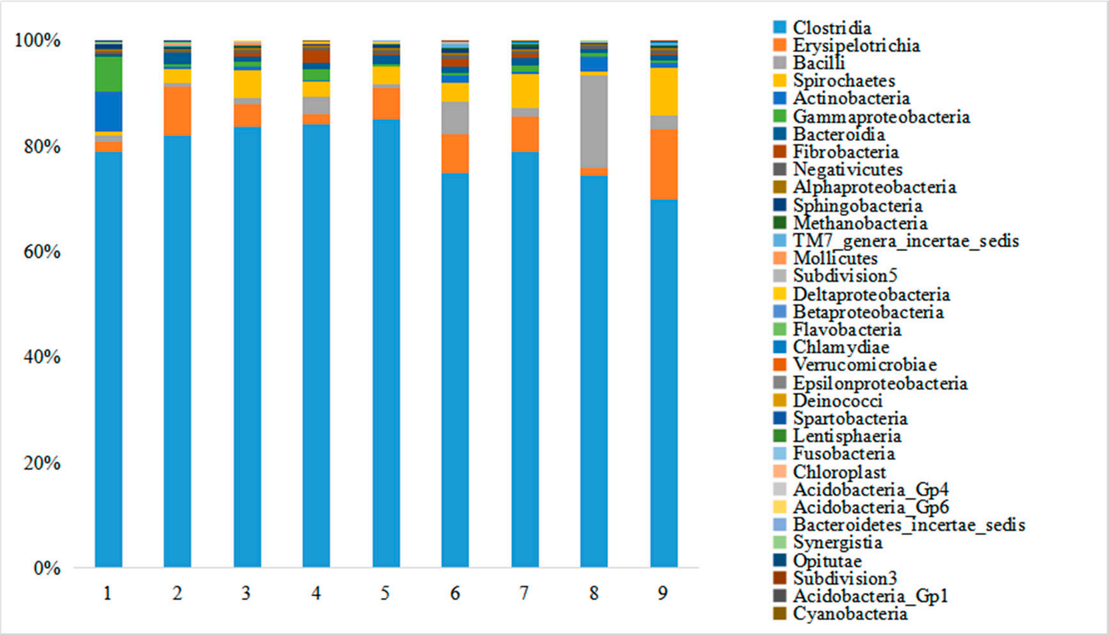

Fig. S2 Class distribution of gut microbiota. Distribution of the class as a percentage of the total number of identified 16S rRNA sequences from fecal samples of the 9 sows.\

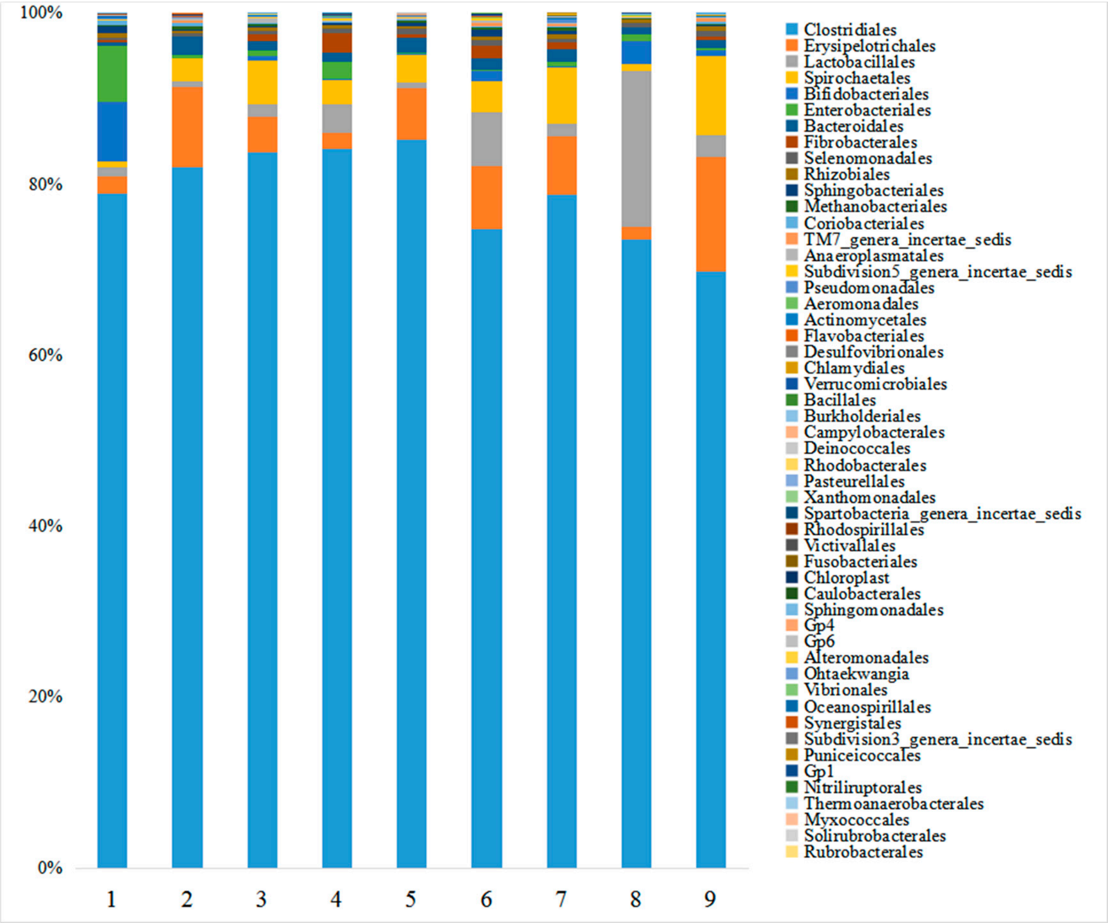

Fig. S3 Order distribution of gut microbiota. Distribution of the order as a percentage of the total number of identified 16S rRNA sequences from fecal samples of the 9 sows.

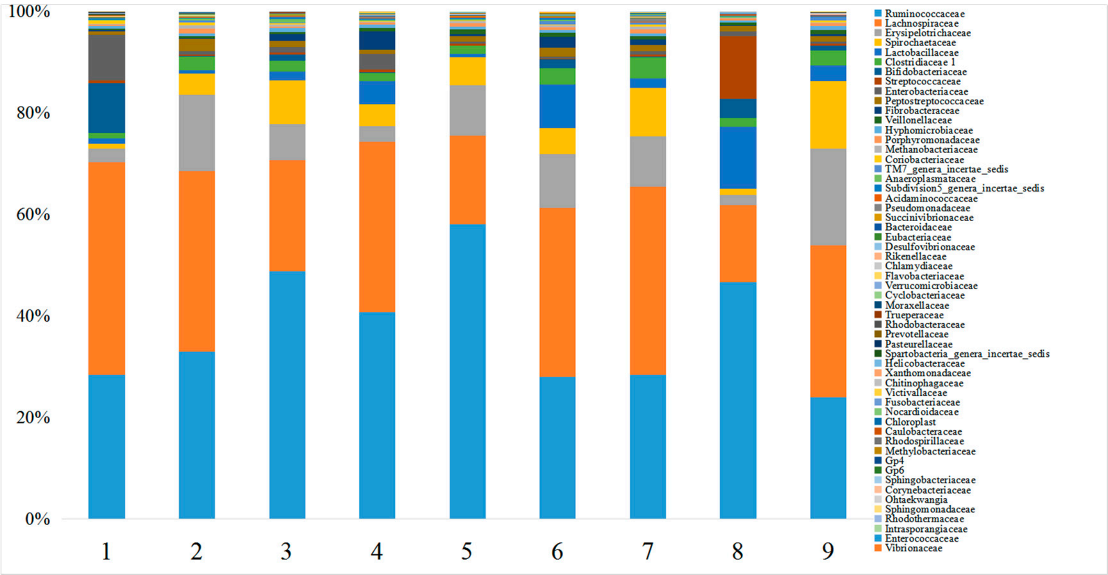

Fig. S4 Family distribution of gut microbiota. Distribution of the family as a percentage of the total number of identified 16S rRNA sequences from fecal samples of the 9 sows.
